# Microscopic polyangiitis presenting with persistent cough and hemoptysis in pediatrics: A case report and review of the literature

**DOI:** 10.3389/fonc.2022.987507

**Published:** 2022-12-06

**Authors:** Yantong Zhu, Xiangrong Zheng

**Affiliations:** Department of Pediatrics, Xiangya Hospital, Central South University, Changsha, China

**Keywords:** microscopic polyangiitis, cough, hemoptysis, children, renal

## Abstract

**Background:**

Microscopic polyangiitis (MPA) is a necrotizing vasculitis that involves small- and medium-sized vessels and is associated with the presence of antineutrophil cytoplasmic antibodies with a perinuclear staining pattern (p-ANCA). The kidney and lungs are the organs primarily affected. MPA is rare in children and is easily misdiagnosed. Below is a complete case history of the course of the disease.

**Case presentation:**

An 11-year-old girl with a 1-month history of cough and hemoptysis showed no improvement after imipenem-cilastatin treatment. p-ANCA and microscopic hematuria and proteinuria were positive, and a chest CT revealed an area of shadow in the bilateral lower lobe of the lungs. Renal biopsies showed crescentic glomerulonephritis, and MPA was diagnosed based on these criteria. The patient exhibited dramatic clinical and imaging improvements after immunosuppressive treatment.

**Conclusion:**

The organs most commonly involved in MPA in children are the lungs, kidneys, skin, nervous system organs, and organs of the gastrointestinal tract. Careful examination should be carried out in these patients while biopsies of the kidney or any other organs remain the gold standard for diagnostic purposes. Pulmonary involvement may be the initial symptom of the disease and should not be confused with pneumonia. A urinalysis should be performed in patients with hemoptysis. Antibiotics should be used with caution.

## Introduction

Antineutrophil cytoplasmic antibody (ANCA)-associated vasculitis (AAV) is a multisystem autoimmune disease that primarily involves small- and medium-sized blood vessels throughout the body. Clinically, it is classified into three types: microscopic polyangiitis (MPA), granulomatosis with polyangiitis (GPA), and eosinophilic GPA (EGPA) (1). Microscopic polyangiitis (MPA) is a necrotizing vasculitis that involves small- and medium-sized vessels, primarily affecting the lungs and kidneys. An analysis of several retrospective adult cases showed that most patients typically present with renal involvement (RI, 80%–100%), cutaneous involvement (CI, 50%), pulmonary involvement (PI, 25%–55%), gastrointestinal involvement (GI, 30%–50%), and nervous system involvement (NSI, 28%) (2). Currently, there are no unified diagnostic criteria for AAV. ANCA positivity is a specific serological marker for AAV, and accurate ANCA testing is important for diagnosis. For example, MPA is associated with the presence of antineutrophil cytoplasmic antibodies with a perinuclear staining pattern (p-ANCA); however, detection of ANCAs is not in itself diagnostic of AAV, and a biopsy remains the gold standard for diagnosis, especially in cases with negative serology or unusual clinical presentation. However, the estimated collective incidence of pediatric vasculitis is about 0.05% (3), and it is even rarer in MPA. We herein describe a complete course of the disease and analyze the clinical features and lab examinations reported for pediatric MPA patients in the past 10 years.

## Case description

**Figure 1:**
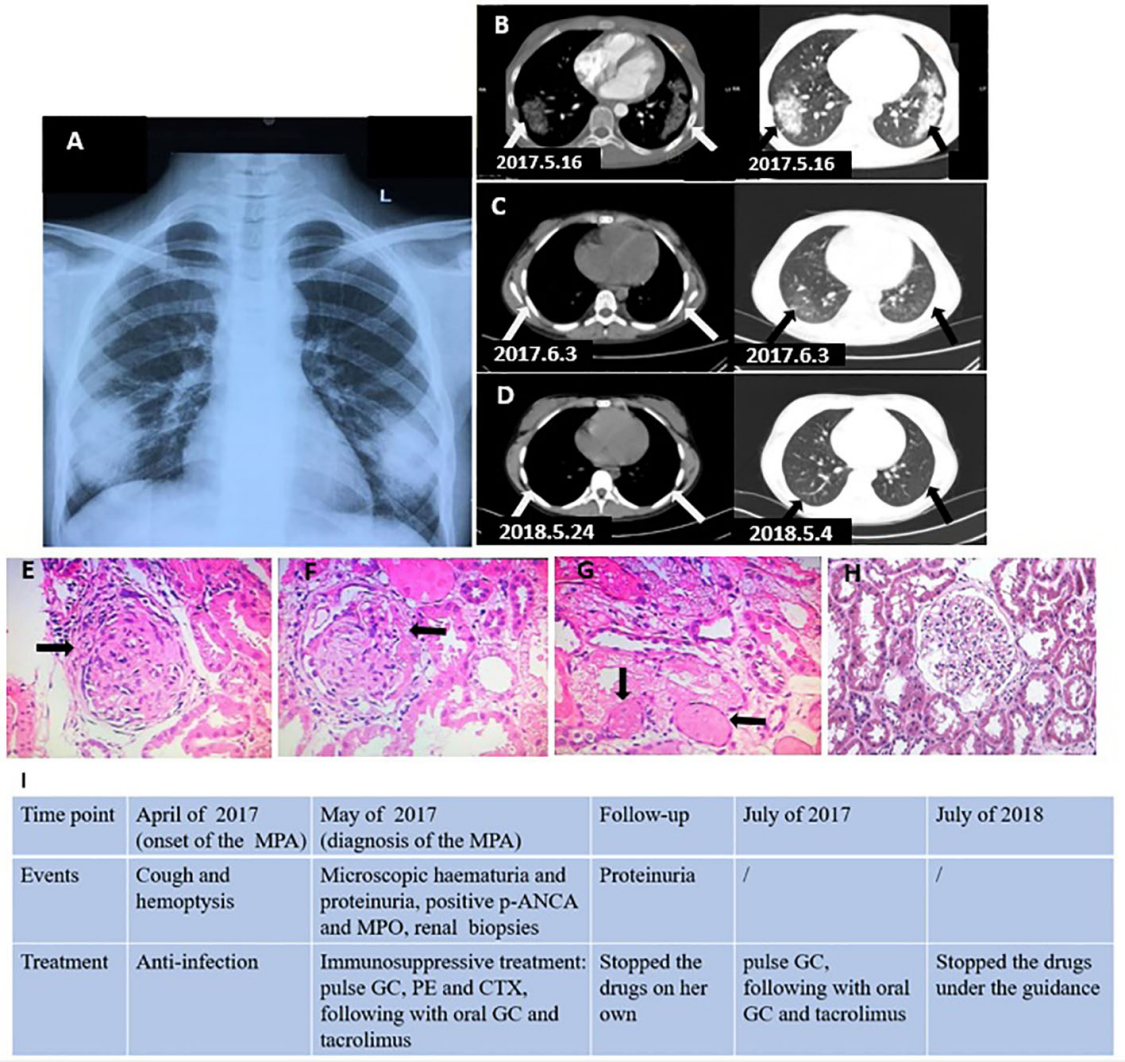
**(A)** X-ray revealed abnormal density areas (arrows) in the bilateral lower lobe of the lungs (symmetric distribution). **(B)** Chest CT revealed a large area of shadow (arrows) in the bilateral lower lobe of the lungs (before the treatment). **(C)** Chest CT revealed the shadows (arrows) in bilateral lungs improved (during the treatment). **(D)** Chest CT revealed the lesions disappeared (after the treatment). **(E, F)** Renal biopsies (HE ×40) revealed glomerulonephritic fibrocellular crescents (arrows). **(G)** Renal biopsies revealed vascular occlusion (arrows). **(H)** Normal control glomerulus. **(I)** Clinical timeline. GC, glucocorticoid; CTX, cyclophosphamide; PE, plasma exchange.

A previously healthy 11-year-old girl (weight, 38.4 kg; height, 155 cm) presented to our department with a 1-month history of cough and hemoptysis. She had an intermittent fever but was without dyspnea, joint pain, headache, or other symptoms. She was initially treated in the local hospital where they suspected infection, and the symptoms improved after 7 days of anti-infection treatment administered on 10 April 2017. The symptoms appeared again 1 month later, and she visited our outpatient department on 10 May 2017. Her parents were healthy and nonconsanguineous. A hemogram reported a leucocyte count of 8.1 × 10^9^/L (neutrophils 52%), platelet count of 407 × 10^9^/L, and hemoglobin count of 106 g/L. The chest X-ray revealed areas of abnormal density (symmetric distribution) in the bilateral lower lobes of the lungs (**Figure 1A**). The patient was diagnosed with lobar pneumonia because of the respiratory symptoms, fever, and X-ray evidence and was treated with parenteral imipenem-cilastatin but had no clinical improvement. Three days later, she was admitted to our hospital to undergo a detailed work-up for the cough and hemoptysis. Upon admission, she had a blood pressure of 96/54 mmHg and a body temperature of 38.5°C. The physical examination on admission was not remarkable. Further examination revealed the following notable laboratory test findings, in chronological order: urinalysis revealed microscopic hematuria (+++) and proteinuria (++) on 14 May; renal function revealed BUN 4.85 mmol/L, Cr 81.6 µmol/L, UA 213.7 µmol/L, and C3 1,300 mg/L; inflammatory markers were slightly increased (ESR 29 mm/h and CRP 19 mg/L); ANA was present in a relatively low titer (1:80), without any antigen-specific antibodies; p-ANCA was positive and MPO-ANCA revealed 50.9 U/ml (normal <20 U/ml); PR3 and GBM were normal on 16 May; the chest CT performed on May 16 revealed an area of dense shadow in the bilateral lower lobes of the lungs, without ground-glass opacity and reticulation (**Figure 1B**), but pulmonary artery CT and PPD were normal. To ultimately establish a proper diagnosis, renal biopsies were performed as a result of the proteinuria and positive MPO and p-ANCA identified on 18 May; finally, the biopsies revealed glomerular cellular and microcellular crescents (**Figures 1E–H**). Immunofluorescence microscopy showed IgA (+) and IgG (++) deposits in the glomerular capillary loop but no C3 and C1q deposits. Based on the respiratory symptoms (cough and hemoptysis), positive MPO and p-ANCA, and the renal biopsies, our patient was finally diagnosed with microscopic polyangiitis (MPA), and so we started immunosuppressive treatment: methylprednisolone (800 mg/day*3 days, two cycles), plasma exchange (1,500 ml/day*4 days), and IV-cyclophosphamide (CTX 800 mg/day once), followed by oral formulation of methylprednisolone (48 mg/day) and tacrolimus (4 mg daily). Methylprednisolone pulse therapy and plasmapheresis resulted in dramatic clinical and imaging improvements (**Figures 1C, D**). The patient stopped the drugs after 2 months with proteinuria (++) and returned to our hospital.

We prescribed methylprednisolone (500 mg/day for 3 days) again, following which she received methylprednisolone (48 mg/day for a month, 24 mg/day for the next month, followed by a gradually reduced dose of 6 mg/day per month, stopping in February 2018) and tacrolimus (4 mg/day, which was gradually reduced (1 mg/day for 3 months) and halted in July 2018). Proteinuria turned negative in September 2017, and the patient continues to have no clinical symptoms, with proteinuria remaining negative. The clinical timeline is presented in **Figure 1I**.

## Discussion and conclusions

AAV is a group of diseases caused by inflammation of the blood vessels. It has a complex pathogenesis. GPA and MPA are associated with a loss of immunological tolerance to PR3 or MPO, whereas EGPA pathogenesis involves two distinct mechanisms that are associated with ANCA-positive and ANCA-negative (4). ANCAs are considered to be central to vasculitis. MPO or PR3 on neutrophil plasma membranes combines with ANCAs that are produced by B cells, which promote the release of destructive proteases, oxygen radicals, as well as autoantigens. Subsequent tissue damage may result in organ dysfunction (4, 5).

Since MPA is rare in children, we initially diagnosed this case with cough and hemoptysis, intermittent fever, and areas of abnormal density in the lungs, such as lobar pneumonia, supported by the fact that the symptoms improved after the first anti-infection treatment. However, further treatment resulted in no improvement. The first anti-infection treatment may have been effective because the infection can cause or worsen MPA when the MPA is not severe. Since there was no further improvement, we then performed urinalysis (hematuria +++, proteinuria ++), MPO-ANCA (50.9 U/ml), and renal biopsies (crescentic glomerulonephritis). Based on the results, MPA was considered. After immunosuppressive treatment, for remission–induction of new-onset organ-threatening or life-threatening MPA, we recommend treatment with a combination of glucocorticoids and cyclophosphamide. In addition, plasma exchange could be used for the treatment of severe diffuse alveolar hemorrhage. The patient’s condition obviously improved, and proteinuria has remained negative. Given the paucity of clinical trials in pediatric ANCA-associated vasculitis, pediatric rheumatologists have relied on adult AAV evidence for management. The European League Against Rheumatism (EULAR) (6) suggests remission–induction of new-onset organ-threatening AAV and recommends a combination of glucocorticoids and either cyclophosphamide or rituximab, and in the case of severe diffuse alveolar hemorrhage, a plasma exchange could be considered for treatment.

### Clinical discussion

Patients with MPA always manifest with multiple system symptoms, such as respiratory symptoms, gastrointestinal symptoms, renal involvement, and nerve system involvement. We recently published a summary of children’s case reports in PubMed (**Supplementary Table**) that showed that the organs involved in MPA in children are the lungs (manifest as dyspnea, cough, and hemoptysis, about 80%), kidneys (manifest as hematuria, proteinuria, and even anuria, about 80%), skin (manifest as purpuric lesions, bullae/hemorrhagic bullae, about 20%), nervous system (manifest as abnormal eye movements, episodes of seizure, and even coma, about 16%), and gastrointestinal tract (manifest as abdominal pain, digestive tract hemorrhage, about 16%). These present as the initial symptoms and affect the prognosis and survival rate. In addition, anemia can also be an initial symptom. Pulmonary involvement, as the initial syndrome, should be carefully distinguished from pneumonia (**Supplementary Table**, the overall usage rate of antibiotics is about 43%, in patients manifesting with pulmonary involvement as the initial syndrome, usage is about 71%), especially in patients who manifest with pulmonary symptoms only. Physical examination always relates to the affected organs, and we should perform further examination in the following order: urinalysis, CRP, and ESR; ANA, ANCA, and chest CT or X-ray; and renal biopsies. We could start immunosuppressive treatment after an MPA diagnosis. There are no unified criteria for the diagnosis of MPA. Granulomatosis polyangiitis (GPA), which is always present with granulomatous inflammation, should be excluded (7, 8). In addition, systemic lupus erythematosus (9), Sjögren syndrome (10), and other connective tissue diseases also involve multiple organs, such as the lungs and kidneys, and we need to distinguish these other systemic diseases from MPA.

### Radiological discussion

The lung is the most commonly affected organ in MPA. Chest CTs show diffuse areas of ground-glass opacity (11), reticulation (12), patchy shadows (13), nodular thickening (14), interstitial pneumonia (15), emphysema (16), and pleural effusion (17). The lesions are always of a symmetric distribution, and all these changes could be alone or coexist in one patient. X-ray and CT findings are inconclusive; MPA can be limited to the lungs. Chest X-rays revealed lesions with an alveolar-filling pattern, which are most often bilateral (2). The imaging manifestations of MPA in the lungs can be easily misdiagnosed as infections or another disease, especially in patients with only lung involvement. Normal X-rays do not mean the lungs are unaffected (18), and most patients will be required to have a chest CT. MPA can also involve the nervous system. Magnetic resonance imaging (MRI) of the brain revealed non-hemorrhagic multiple lesions (13), reversible posterior leukoencephalopathy syndrome (16), and even hemorrhagic stroke (19). We recommend that brain MRI be performed on patients diagnosed with, or suspected of having, MPA.

### Pathological discussion

Biopsies of the kidney or any other organ remain the gold standard for diagnostic purposes (6). Generally, renal biopsies can reveal pathological changes such as necrotizing glomerulonephritis (18), cellular and microcellular crescent glomerulonephritis, and sclerosed glomerulonephritis (12). Immunofluorescence microscopy demonstrates pauci-immune glomerulonephritis, and slight IgA deposits have been reported (20). In addition to the confirmation of MPA diagnosis, a renal biopsy also can help the clinician to provide a renal prognosis of the disease.

In summary, MPA is an unusual disease in children involving multiple systems. The clinical symptoms vary, but the organs most involved in MPA in children are the lungs, kidneys, skin, nervous system, and gastrointestinal tract. A thorough examination should be carried out in these patients, with biopsies of the kidney or any other organs remaining the gold standard for diagnostic purposes. Pulmonary involvement should be carefully distinguished from pneumonia; urinalysis should be taken in those patients with hemoptysis; and finally, antibiotics should be used with caution.

## Data availability statement

The original contributions presented in the study are included in the article/**Supplementary Material**. Further inquiries can be directed to the corresponding authors.

## Ethics statement

The studies involving human participants were reviewed and approved by Ethics committee of Xiangya Hospital, Central South University Approval Documents for Scientific Research Projects (No. 2018121104). Written informed consent to participate in this study was provided by the participants’ legal guardian/next of kin. Written informed consent was obtained from the individual(s), and minor(s)’ legal guardian/next of kin, for the publication of any potentially identifiable images or data included in this article.

## Author contributions

All authors read and approved the final manuscript. The first draft of the manuscript was produced by YZ and XZ. All authors contributed to the article and approved the submitted version.

## References

[1] Zhao WM Wang ZJ Shi R Zhu YY Zhang S Wang RF. Environmental factors influencing the risk of ANCA-associated vasculitis. Front Immunol (2022) 13:991256. doi: 10.3389/fimmu.2022.991256 36119110 PMC9479327

[2] Villiger PM Guillevin L. Microscopic polyangiitis: Clinical presentation. Autoimmun Rev (2010) 9(12):812–9. doi: 10.1016/j.autrev.2010.07.009 20656070

[3] Eleftheriou D Batu ED Ozen S Brogan PA. Vasculitis in children. Nephrol Dial Transplant (2015) 30 Suppl 1:i94–103. doi: 10.1093/ndt/gfu393 25550447

[4] Kitching AR Anders HJ Basu N Brouwer E Gordon J Jayne DR. ANCA-associated vasculitis. Nat Rev Dis Primers (2020) 6(1):71. doi: 10.1038/s41572-020-0204-y 32855422

[5] Trivioli G Marquez A Martorana D Tesi M Kronbichler A Lyons PA. Genetics of ANCA-associated vasculitis: role in pathogenesis, classification and management. Nat Rev Rheumatol (2022) 18(10):559–74. doi: 10.1038/s41584-022-00819-y 36109667

[6] Yates M Watts RA Bajema IM Cid MC Crestani B Hauser T. EULAR/ERA-EDTA recommendations for the management of ANCA-associated vasculitis. Ann Rheum Dis (2016) 75(9):1583–94. doi: 10.1136/annrheumdis-2016-209133 27338776

[7] Jiang B Zhao YY Wei SH. Granulomatosis with polyangiitis: the relationship between ocular and nasal disease. Ocul Immunol Inflammation (2013) 21(2):115–8. doi: 10.3109/09273948.2012.747618 23252657

[8] Jennette JC Falk RJ Bacon PA Basu N Cid MC Ferrario F. 2012 revised international chapel hill consensus conference nomenclature of vasculitides. Arthritis Rheum (2013) 65(1):1–11. doi: 10.1002/art.37715 23045170

[9] Zhang R Dang X Shuai L He Q He X Yi Z. Lupus erythematosus panniculitis in a 10-year-old female child with severe systemic lupus erythematosus: A case report. Med (Baltimore) (2018) 97(3):e9571. doi: 10.1097/MD.0000000000009571 PMC5779747 29504978

[10] Li H Xiong Z Liu J Li Y Zhou B. [Manifestations of the connective tissue associated interstitial lung disease under high resolution computed tomography]. Zhong Nan Da Xue Xue Bao Yi Xue Ban (2017) 42(8):934–9. doi: 10.11817/j.issn.1672-7347.2017.08.010 28872085

[11] Brunner J Freund M Prelog M Binder E Sailer-Hoeck M Jungraithmayr T. Successful treatment of severe juvenile microscopic polyangiitis with rituximab. Clin Rheumatol (2009) 28(8):997–9. doi: 10.1007/s10067-009-1177-0 19390907

[12] Jindal G Cruz SD Punia RP Kaur R. Refractory anemia as a presenting feature of microscopic polyangiitis: a rare vasculitis in children. Indian J Pediatr (2011) 78(10):1287–9. doi: 10.1007/s12098-011-0459-0 21630073

[13] Wang S Habib S Umer S Reisman L Raman V. Recurrent posterior reversible encephalopathy syndrome in a child with microscopic polyangiitis. J Clin Rheumatol (2015) 21(2):113–4. doi: 10.1097/RHU.0000000000000222 25710870

[14] Alpigiani MG Calcagno A Salvati P Rossi GA Barbano G Ghiggeri G. Late onset of pANCA renal and pulmonary vasculitis in a girl affected by undifferentiated connective tissue disease. Lupus (2010) 19(5):655–7. doi: 10.1177/0961203309349740 20133348

[15] Roszkiewicz J Smolewska E. From fibrosis to diagnosis: a paediatric case of microscopic polyangiitis and review of the literature. Rheumatol Int (2018) 38(4):683–7. doi: 10.1007/s00296-017-3923-y 29294176

[16] Bhadu D Kumar P Malhotra KP Sharma A Sharma M Srivastava D. Central nervous system vasculitis in pediatric microscopic polyangiitis. Acta Reumatol Port (2016) 41(4):372–5. 27156236

[17] Wang H Sun L Tan W. Clinical features of children with pulmonary microscopic polyangiitis: report of 9 cases. PloS One (2015) 10(4):e0124352. doi: 10.1371/journal.pone.0124352 25923706 PMC4414499

[18] Dziuban EJ Castle VP Haftel HM. Microscopic polyangiitis in an adolescent presenting as severe anemia and syncope. Rheumatol Int (2011) 31(11):1507–10. doi: 10.1007/s00296-009-1270-3 20013269

[19] Iglesias E Eleftheriou D Mankad K Prabhakar P Brogan PA. Microscopic polyangiitis presenting with hemorrhagic stroke. J Child Neurol (2014) 29(8):NP1–4. doi: 10.1177/0883073813488661 23690295

[20] Kaseda K Marui Y Suwabe T Hoshino J Sumida K Hayami N. Kidney transplantation for a patient with refractory childhood-onset ANCA-associated vasculitis. Mod Rheumatol (2016) 26(2):307–9. doi: 10.3109/14397595.2013.877327 24645722

