# Corrigendum: Microscopic polyangiitis presenting with persistent cough and hemoptysis in pediatrics: A case report and review of the literature

**DOI:** 10.3389/fonc.2023.1150079

**Published:** 2023-02-14

**Authors:** Yantong Zhu, Xiangrong Zheng

**Affiliations:** Department of Pediatrics, Xiangya Hospital, Central South University, Changsha, China

**Keywords:** microscopic polyangiitis, cough, hemoptysis, children, renal

**Error in Author List**

In the published article, there was an error in the author list, and both Yantong Zhu and Xiangrong Zheng were erroneously listed as corresponding authors. The corrected corresponding author list appears below.

Xiangrong Zheng

zxr_168@126.com

The authors apologize for this error and state that this does not change the scientific conclusions of the article in any way. The original article has been updated.

