# Supplementary material for: Microscopic polyangiitis presenting with persistent cough and hemoptysis in pediatrics: A case report and review of the literature
Source: Front Oncol. 2022 Dec 6;12:987507. doi: 10.3389/fonc.2022.987507 (PMC9763891; doi:10.3389/fonc.2022.987507)
Supplement: Supplementary file 1 [file Table_1.docx]

supplementary table： Clinical features and lab examination of the MPA patients in recent years

|  | Di. Maria, M. V.et al. （1） | Brunner, J. et al. （2） | Dziuban, E. J. et al. （3） | HaradaT. et al.（4） | Jindal, G et al.（5） | Yamato, K et al.（6） | Yamada, Y et al.（7） | Iglesias, E et al.（8） | Wang, H et al.（9） | Wang, S et al.（10） | Adachi, A et al.（11） | Bhadu Det al.（12） | Kaseda, K et al.（13） | Luis FF et al.（14） | Pilania, R. K.et al.（15） | Roszkiewicz, J（16） |
| --- | --- | --- | --- | --- | --- | --- | --- | --- | --- | --- | --- | --- | --- | --- | --- | --- |
| Age(y) | 13 | 14 | 16 | 13 | 9 | 11 | 7 | 12 | 1.9-16.2 | 10 | 11 | 14 | 12 | 16 | 2 | 6 |
| Sex | M | F | M | F | F | F | F | F | 7F/2M | F | F | F | F | F | F | F |
| Clinical manifestation  PI  RI  CI  NSI  GI  Others | +  +  -  -  -  - | +  +  +  -  -  Arthritis  Anemia | +  +  -  -  -  Anemia  hypertension | -  +  -  -  +  - | +  +  -  -  -  Anemia  joint pain | -  +  -  -  -  Anemia | +  +  +  -  -  Anemia | -  -  -  +  +  - | 9/9  7/9  -  -  2/9  Arthritis  Anemia | +  +  +  +  -  - | +  +  +  -  -  Arthritis | +  +  +  +  -  Arthritis | -  +  -  -  -  - | +  +  -  +  - | +  -  -  -  -  pulmonary hypertension | +  +  -  -  -  - |
| Initial syndromes | PI | CI | PI | RI | Anemia | Anemia | Anemia | NSI | 8/9PI,2/9RI | CI+PI | CI | Arthritis | RI | PI | PI | PI |
| MPO/p-ANCA | + | + | + | + | + | + | + | + | 9/9 | + | + | + | + | + | + | + |
| X | + | N/A | - | N/A | N/A | + | + | - | N/A | N/A | + | + | N/A | N/A | + | + |
| CT  ground-glass opacity  reticulation  emphysema  patchy shadows  Interstitial pneumonia  pleural effusion  nodules  Mosaic perfusion | N/A | +  -  -  -  -  -  +  - | +  -  -  -  -  -  +  - | N/A | +  +  -  -  -  -  -  - | +  -  -  -  -  -  -  - | +  -  -  -  -  -  -  - | N/A | 1/9 -  5/9  -  -  3/9  1/9  1/9  -  - | +  -  -  +  -  -  -  - | N/A | +  +  +  -  -  -  -  - | N/A | - | -  -  -  -  -  -  -  + | -  -  -  -  +  -  -  - |
| Renal Biopsy | N/A | + | + | + | + | + | - | N/A | 7/9 | + | N/A | + | + | N/A | N/A | + |
| Misdiagnosis | - | - | - | - | - | - | - | - | 4/9 IPH | Pneumonia | - | TB | - | Infection | - | Pneumonia |
| Antibiotic | + | - | - | - | - | - | + | - | N/A | + | - | + | - | + | - | + |

M, male; F, Female; PI, Pulmonary Involvement; RI, Renal Involvement; CI, Cutaneous Involvement; NSI, Nervous System Involvement; +,positive; -,negative; p-ANCA/MPO, perinuclear ANCA/myeloperoxidase; N/A, not applicable; X, X-rays; CT, [computerized tomography](../../../Program%20Files%20(x86)/Dict/7.5.0.0/resultui/dict/); IPH, idiopathic pulmonary hemosiderosis

1. Di Maria, M.V., R. Hollister and J. Kaufman, *Case report: severe microscopic polyangiitis successfully treated with extracorporeal membrane oxygenation and immunosuppression in a pediatric patient.* Curr Opin Pediatr, 2008. **20**(6): p. 740-2.

2. Brunner, J., M. Freund, M. Prelog, E. Binder, M. Sailer-Hoeck, T. Jungraithmayr, et al., *Successful treatment of severe juvenile microscopic polyangiitis with rituximab.* Clin Rheumatol, 2009. **28**(8): p. 997-9.

3. Dziuban, E.J., V.P. Castle and H.M. Haftel, *Microscopic polyangiitis in an adolescent presenting as severe anemia and syncope.* Rheumatol Int, 2011. **31**(11): p. 1507-10.

4. Harada, T., S. Ito, T. Sasaki, R. Kunisaki, H. Shiojima, M. Ogawa, et al., *GI involvement of sigmoid mucosal erosion in a 13-year-old girl with microscopic polyangiitis.* Gastrointest Endosc, 2011. **74**(4): p. 937-9.

5. Jindal, G., S.D. Cruz, R.P. Punia and R. Kaur, *Refractory anemia as a presenting feature of microscopic polyangiitis: a rare vasculitis in children.* Indian J Pediatr, 2011. **78**(10): p. 1287-9.

6. Yamato, K., T. Ishii and T. Kawamura, *Microscopic polyangiitis in a girl with severe anemia and no respiratory symptoms.* Pediatr Int, 2012. **54**(4): p. 541-3.

7. Yamada, Y., C. Kitagawa, I. Kamioka, K.R. Chen and M. Oka, *A case of microscopic polyangiitis with skin manifestations in a seven-year-old girl.* Dermatol Online J, 2013. **19**(9): p. 19624.

8. Iglesias, E., D. Eleftheriou, K. Mankad, P. Prabhakar and P.A. Brogan, *Microscopic polyangiitis presenting with hemorrhagic stroke.* J Child Neurol, 2014. **29**(8): p. NP1-4.

9. Wang, H., L. Sun and W. Tan, *Clinical features of children with pulmonary microscopic polyangiitis: report of 9 cases.* PLoS One, 2015. **10**(4): p. e0124352.

10. Wang, S., S. Habib, S. Umer, L. Reisman and V. Raman, *Recurrent posterior reversible encephalopathy syndrome in a child with microscopic polyangiitis.* J Clin Rheumatol, 2015. **21**(2): p. 113-4.

11. Adachi, A., M. Komine, S. Murata, H. Tsuda, Y. Kawahara, A. Morimoto, et al., *Pediatric case of microscopic polyangiitis with skin manifestations resembling vesiculobullous type erythema elevatum diutinum with immunoglobulin A antineutrophil cytoplasmic antibody.* J Dermatol, 2016. **43**(11): p. 1377-1378.

12. Bhadu, D., P. Kumar, K.P. Malhotra, A. Sharma, M. Sharma and D. Srivastava, *Central nervous system vasculitis in pediatric microscopic polyangiitis.* Acta Reumatol Port, 2016. **41**(4): p. 372-375.

13. Kaseda, K., Y. Marui, T. Suwabe, J. Hoshino, K. Sumida, N. Hayami, et al., *Kidney transplantation for a patient with refractory childhood-onset ANCA-associated vasculitis.* Mod Rheumatol, 2016. **26**(2): p. 307-9.

14. Flores-Suarez, L.F., M.A. Alba and G. Tona, *Severe microscopic polyangiitis with unilateral vocal cord paralysis as initial manifestation.* Colomb Med (Cali), 2017. **48**(1): p. 32-34.

15. Pilania, R.K., S.R. Dhawan, J.L. Mathew, S. Singh, K.S. Sodhi and M. Singh, *ANCA-associated Vasculitis Presenting as Severe Pulmonary Hypertension and Right Heart Failure.* Indian J Pediatr, 2017. **84**(10): p. 799-801.

16. Roszkiewicz, J. and E. Smolewska, *From fibrosis to diagnosis: a paediatric case of microscopic polyangiitis and review of the literature.* Rheumatol Int, 2018. **38**(4): p. 683-687.
